# A free-standing, phase-change liquid metal mold for 3D flexible microfluidics

**DOI:** 10.3389/fbioe.2022.1094294

**Published:** 2022-12-05

**Authors:** Sheng Yan, Qingwei Yuan, Jialin Wu, Zixuan Jia

**Affiliations:** ^1^ Institute for Advanced Study, Shenzhen University, Shenzhen, China; ^2^ Nanophotonics Research Center, Institute of Microscale Optoelectronics, Shenzhen University, Shenzhen, China

**Keywords:** flexible microfluidics, microfabrication, lab-on-a-chip, liquid metal, viscoelastic manipulation

## Abstract

This paper describes a method to fabricate the 3D microfluidic channel using the free-standing, phase-change gallium mold. Three approaches to prepare the free-standing gallium molds are described. The solid metal framework is strong enough to stand against the gravity. After casting, the embedded gallium molds are melted from solid to liquid and then extracted from the encasing elastomer to form the 3D microfluidic channel due to the phase change property. Since this method is compatible with many encasing materials (e.g., elastomers, gels, resins, ceramics), the encasing materials will bring novel functionalities to the microfluidic chip. Two proof-of-concept experiments have been demonstrated. Firstly, a soft, sticky, on-skin microfluidic cooler is developed based on this method to deliver the focused, minimal invasive cooling power at arbitrary skins of human body with temperature control. Secondly, an ultra-stretchable viscoelastic microchannel with the ultra-soft base is fabricated to continuously tune the viscoelastic particle focusing with a large dynamic range. This proposed technique suggests the new possibilities for the development of lab-on-a-chip applications.

## 1 Introduction

The 3D fabrication technologies flourish the development of 3D microfluidics (Su et al., 2020) and enables the new applications in 3D organ chips (Dornhof et al., 2022) and advanced microfluidic networks (Luan et al., 2021). There are several strategies to construct the 3D microstructures for microfluidic channel including lithography, laser writing, colloidal assembly and direct-write techniques. (Cumpston et al., 1999; Campbell et al., 2000; Therriault et al., 2003; Gratson et al., 2004) However, most work has focused on the construction of polymers. Since these polymers are rigid to stand the designed structures, they are not soft enough to satisfy the demand of flexible microfluidics (e.g., epidermal microfluidic devices (Reeder et al., 2019) and microfluidic electronics (Zhang et al., 2022)).

Smart methods to construct the liquid frameworks for fabricating microfluidic channel were developed. The solid metal wires (e.g., tungsten wire) were used to construct the 3D molds. (Li and Xu, 2015) After encasing, the metal wires will be physically pulled from the encasing materials. The 1D, straight channel can be reconfigured into complex microchannels by the secondary manipulation. Although the solid metal wires are strong enough to be intactly extracted from the elastomers, the microchannel may be destroyed during the extraction of metal wires. In addition to the solid metals, the gallium-based liquid metals (LMs) keep in liquid phase at the room temperature and their oxide skin on the surface allows for the formation of mechanically stable structures. Dickey et al. (2008); (Tang et al., 2021) (Ladd et al., 2013) reported a direct-write method to pattern the 3D, free-standing LM structures. Later, this method was further applied to fabricate double spiral channels with a semicircular cross-section. (Parekh et al., 2016) Yan et al. (2018); (Hu et al., 2021) developed an amalgamation-assisted lithography where LMs are pattern on the masked copper tape. The various unconventional microchannels are developed by the LM molds. Khoshmanesh et al. (Nguyen et al., 2019) used the LMs as fugitive ink to generate the microdomes in the microchannel. Although the LMs are feasible to fabricate the microfluidic channels, the LMs cannot form the long wires to satisfy the demand of out-of-plane, complex microchannels due to the Rayleigh instability (Kull, 1991). Owing to the supercooling property, the LMs can be frozen at low temperature and melted at room temperature. Majidi et al. (Fassler and Majidi, 2013) injected the EGaIn into the microchannel, froze EGaIn LMs, and removed the 3D LM microstructures from the mold. Although the 3D LM microstructures were manipulated and encapsulated by the elastomer, the 3D LM microstructures could only operate on the cold plate due to the low melting point (15°C). Furthermore, this 3D LM microstructure is not suitable for preparing the microfluidic channel because the LM microstructure will melt when PDMS cures at room temperature.

In this work, we use LM (Galium) wire as the sacrificial material to create the 3D microfluidic channel. The phase change gallium can transition from solid to liquid when the temperature is over 29.4°C. In the solid phase, the gallium wire can be programmed into various free-standing structures due to the stiffness of the solid gallium, while the liquid gallium can easily be extracted from the elastomers to form the 3D microfluidic channel without the damage of the microchannel. Two methods to create the free-standing LM mold are developed and encasing the free-standing LM mold into various elastomers are demonstrated. As two proof-of-concept applications, we create the flexible microfluidic channels using the free-standing LM molds as a soft, wearable cooler and an ultra-stretchable viscoelastic channel.

## 2 Results and discussion

### 2.1 Working mechanism

**FIGURE 1:**
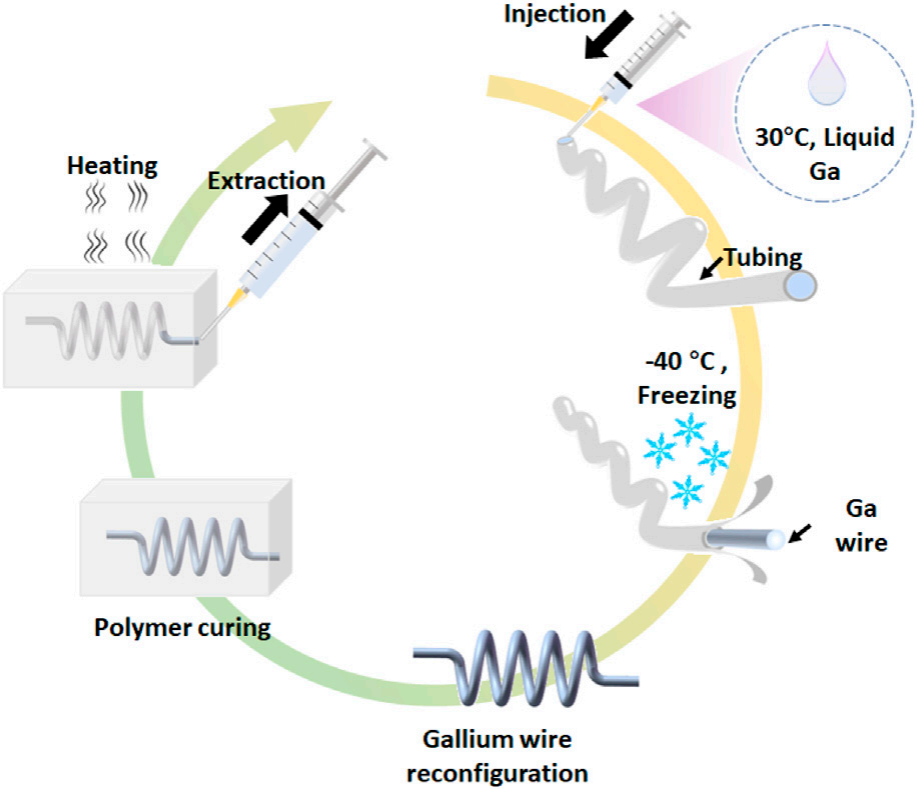
Schematic of the fabrication of 3D microfluidic devices using the free-standing liquid metal molds.

Figure 1 depicts the creation of the free-standing gallium wire mold and 3D microfluidic channel. The melted gallium is loaded in the syringe and injected into the silicone tubing (the inner diameter of 250 μm or 350 μm). Due to the supercooling effect, the gallium has the freezing point of −40°C and melting point of 29.4°C. Under −40°C, the liquid gallium in the tubing turns solid. The tubing is then dissected to obtain the gallium wire. The gallium wire can be reconstructed into 3D structures at the room temperature (25°C). The 3D, free-standing gallium wire molds as the sacrificial material are encased in the elastomers and then melted at the temperature of over 30°C. The encased liquid gallium was washed by the 0.1 M NaOH for serval times. Finally, the 3D microfluidic channel is obtained. The diameter of gallium wire is dependence of the inner diameter of silicone tubes. With different diameters of silicone tubes, we can prepare the gallium wire with the diameter ranging from 120 μm to 350 μm (see Supplementary Figure S1). However, the resolution of the gallium wire can be further improved by the precise micromachining of the gallium wire. In the following experiments, we use the gallium wire with the diameter of 250 μm for preparing the 3D, free-standing microstructures, unless otherwise stated.

### 2.2 Three approaches to fabricate the free-standing LM molds

The 3D, free-standing LM molds can be developed using at least three approaches: (1) Assembly of gallium wire *via* low-temperature welding, (2) Winding gallium wire, and (3) Hybrid method that combines welding assembly and winding.

**FIGURE 2:**
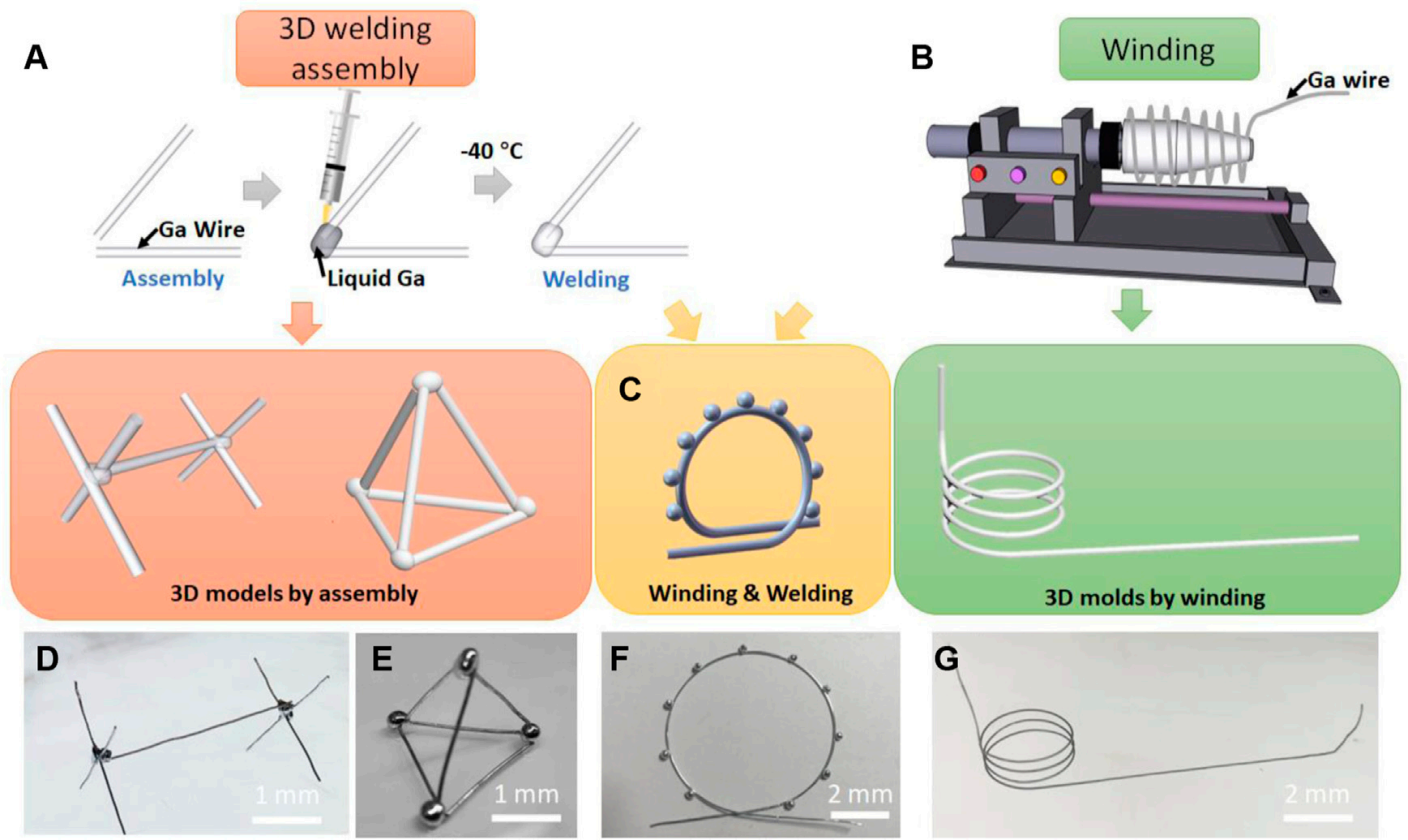
Creation of LM molds. **(A)** 3D LM microstructures by welding. **(B)** 3D LM microstructures by winding. **(C)** 3D LM microstructures by a hybrid method that combines the welding and winding. **(D)** A free-standing LM bridge. **(E)** A free-standing LM tetrahedron. **(F)** A LM “Ferris wheel”. **(G)** A 3D spiral structure.

Figures 2A–E show the structures constructed by the welding. The gallium wires are put together and the liquid gallium droplet is applied at the joint. After liquid gallium freezing at −40°C, the neighboring gallium wires are assembled by the low-temperature welding. This step can be repeated many times to obtain the complex free-standing LM molds. This process can fabricate 3D microstructures of LM such as bridge and tetrahedron.

Figures 2B, G show the structures constructed by winding of gallium wire. The gallium wire is plastically deformed by the automatic winding machine to form the 3D spiral structures. The diameter and pitch of the 3D spiral microstructures can be modulated by controlling the rotation system. In addition, the hybrid method that combines 3D assembly and winding is able to fabricate the more complex 3D structures. The structure in Figure 2c and 2f is an example formed in the hybrid manner. The gallium wire was first plastically deformed on the automatic winding machines to obtain the circular structure. Second, the liquid gallium was evenly dropped on the circular structure. After freezing of the gallium droplets, a “Ferris wheel” structure is formed. Using this hybrid method, we can also prepare the microchannel mold with multiple branches and turn the mold from 2D to 3D structures (see Supplementary Figure S2).

### 2.3 Fabrication of 3D microchannels

**FIGURE 3:**
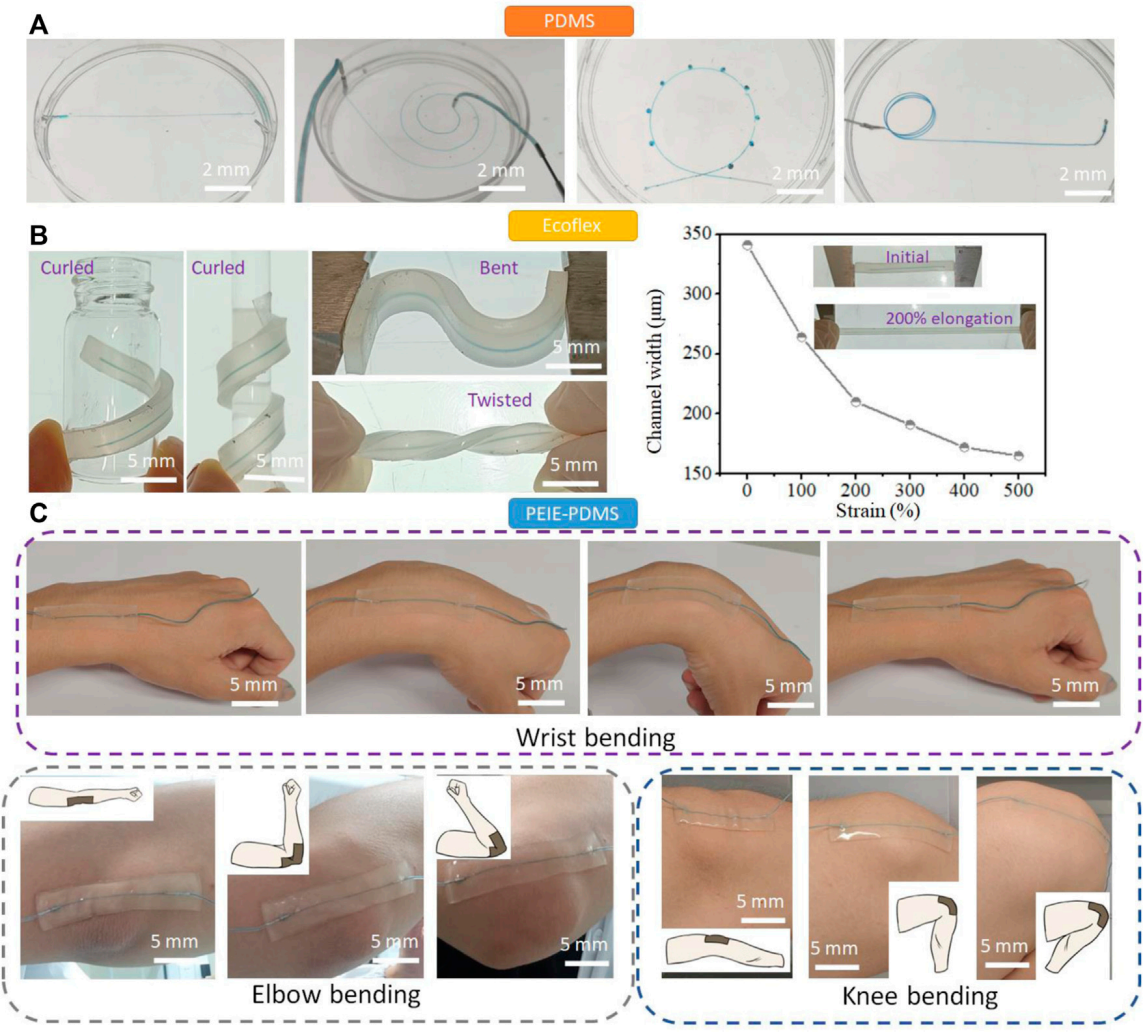
Creation of the microfluidic channels. **(A)** The various PDMS microchannel including straight channel, spiral channel, “Ferris wheel” channel, and 3D helical channel fabricated *via* the LM molds. **(B)** Soft, stretchable Ecoflex microchannels fabricated using the LM molds. The Ecoflex microfluidic chips can be curled, bent, twisted, and elongated to modulate the structure and size of the microchannel. **(C)** The soft, sticky PEIE-PDMS microfluidic chip fabricated using the LM molds. The prepared PEIE-PDMS microchip can have a conformal contact with human joints such as wrist, elbow, and knee.

With the advanced production method to generate the free-standing LM molds, the various 3D microchannel can be created. Since polydimethylsiloxane (PDMS) is a widely used material for the fabrication of microfluidic channel, we first embedded the LM molds in PDMS (Figure 3A). Since the LM molds are solid, they can keep its original structure during PDMS pouring and curing. The microchannels such as straight channel, spiral channel, “Ferris wheel” channel, and 3D helical channel have been demonstrated using this process. The microchannel can be generated as the whole using this process without the chemical bonding, which can significantly reduce the flow leakage.

Ecoflex is a soft, strong, and ultra-stretchable elastomer, bringing new possibilities in the field of flexible electronics (Xu et al., 2019) and soft robotics (Polygerinos et al., 2017). Since Ecoflex does not easily bond to itself by plasma surface treatment after curing, Ecoflex have not been used to prepare the microfluidic channel. Here, we embedded the gallium wires in Ecoflex to form the Ecoflex microchannel (Figure 3B). The mechanical properties of Ecoflex turn the microchannel soft and stretchable. The Ecoflex channel can be curled, bent, and twisted, turning the 1D channel (straight channel) into the 3D channel. Unlike PDMS with the fracture strain of ∼70%, the fracture strain of Ecoflex is ∼600%. (Li et al., 2022) When the Ecoflex matrix is elongated from 0 to 500%, the internal microchannel in Ecoflex is dynamically modulated from 345 μm to 159 μm. The dynamic modulation of Ecoflex microfluidic channel reduces the production cycle of the fabrication of microfluidic channel and enables and the wide-range manipulation of cells.

Polyethylenimine (PEIE)-PDMS is a soft, stretchable, and sticky elastomers, which has been used for epidermal electronics. (Jeong et al., 2016) We use the sticky PEIE-PDMS elastomer to encase the LM molds and turn the conventional PDMS microchannel to the wearable microfluidic devices. The PEIE-PDMS is prepared by adding 20 μL PEIE solution to 1 g PDMS mixture (PDMS base: crosslinker = 10:1). The prepared PEIE-PDMS microfluidic devices can be stuck on the body joints such as wrist, elbow, and knee (Figure 3C). The movement of body joints normally generates the large strain on the wearable microfluidic device. No delamination from the skin of the wrist, elbow, and knee was observed during movement, showing the strong adhesion of this material (adhesion force ∼0.8N/cm). We can also attach the straight channel to the human fist, which can generate the 3D microchannel (see Supplementary Figure S3). Taking advantage of the sticky PDMS-based elastomer, we can fabricate the modular microfluidics by stacking the PEIE-PDMS channel. Supplementary Figure S4 shows that two PEIE-PDMS microchannels are stacked to form a new 3D microchannel. The liquid was pumped into the bottom layer of the microchannel, and then passed the top channel. Due to the adhesion of two layers, no liquid was leaked at the junction. More layers can be stacked to form the more complex microfluidic network and microfluidic elements such as pumps, valves, mixers, separators, and flow regulators can be combined together to generate the advanced microfluidic systems using this process.

The free-standing LM molds can be encased with elastomers, polymers and ceramics. These encasing materials bring the new functionalities to the 3D microfluidic channel. Ecoflex brings the ultra-stretchability to the 3D microfluidic devices, while PEIE-PDMS opens a new window for wearable microfluidics due to the strong adhesion. Moreover, the encasing materials can be temperature-responsive, pH-responsive, magnetically-responsive, and electrically-responsive polymers, enabling the 3D microfluidic devices turn into the 4D microfluidic devices under the external stimuli. (Wang et al., 2019; Yun et al., 2019; Zhang et al., 2020)

### 2.4 Soft, sticky, on-skin microfluidic cooler

**FIGURE 4:**
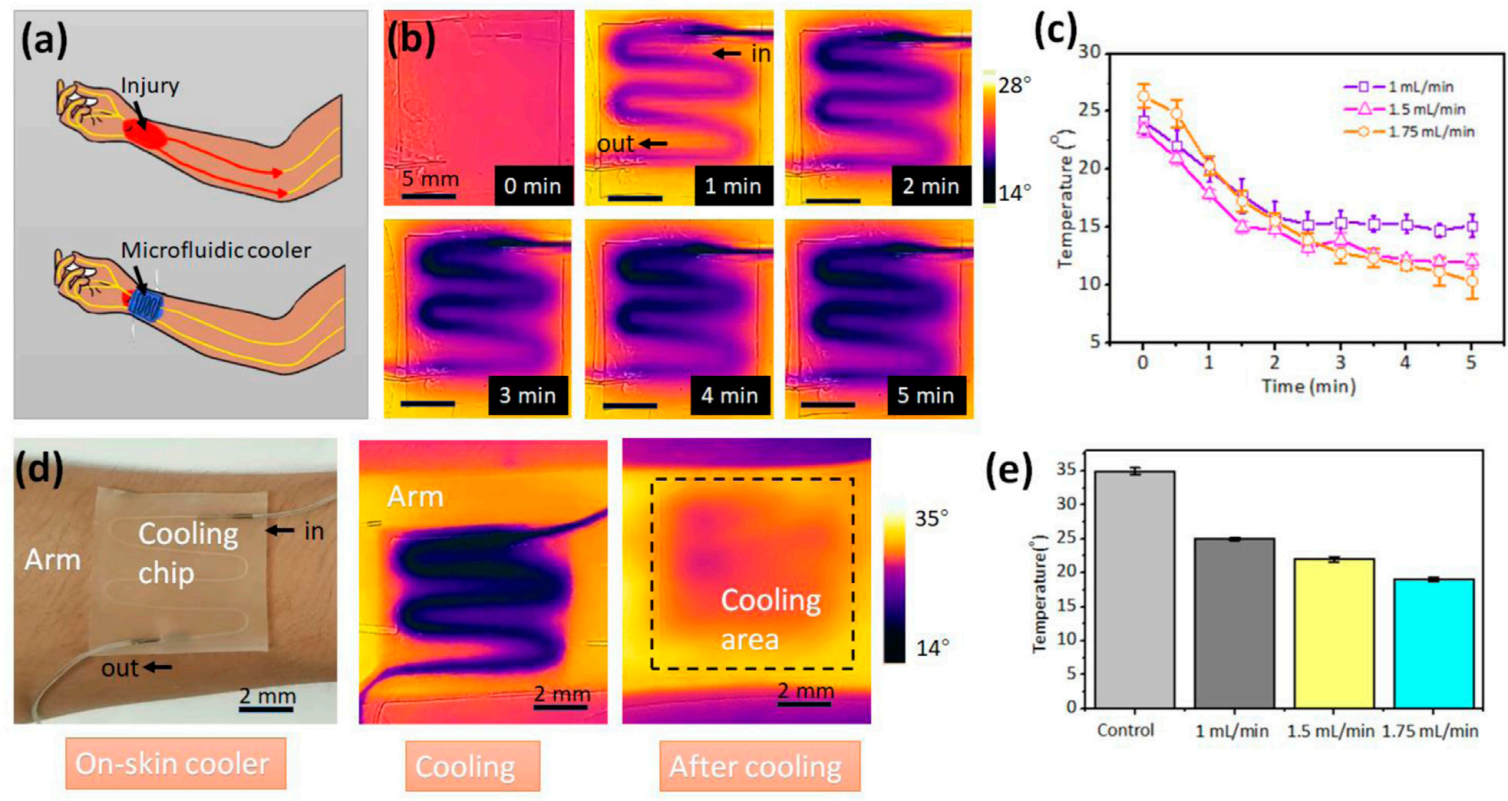
Wearable microfluidic cooler. **(A)** Schematic of the microfluidic cooler that can block the peripheral nerve activity during the pain relief. **(B)** Infrared thermal images of the microfluidic cooler. **(C)** The relationship between flow rate of the cold water and the surface temperature of the cooler. The obtained temperature is the average temperature by analyzing the infrared thermal images. **(D)** The microfluidic cooler sticking on the human arm for local cooling. **(E)** The skin temperatures at the various flow rate of cold water.

After injury due to an accident or medical surgery, local cooling can block the peripheral nerve activity, representing an attractive form for pain relief. (Reeder et al., 2022) However, traditional cooling technologies are in rigid form and imprecise cooling, and may cause secondary pain due to the cold. Here, we demonstrate a soft, wearable, on-skin microfluidic cooler comprised of a serpentine channel and PEIE-PDMS elastomer (Figure 4A). The cold water flowing in the serpentine channel brings the heat from the skin, while the soft, sticky PEIE-PDMS elastomer enables the conformal contact with the skin. This microfluidic cooler enables delivery of focused, minimal invasive cooling power at arbitrary skins of human body with temperature control.

The cold water (0°C) was injected into the channel at the flow rate 1 ml/min. Figure 4B shows the surface temperature of the microfluidic cooler with the increasement of time. Figure 4C depicts the relationship between the flow rates of cold water and surface temperature of the microfluidic cooler. The temperature is the average temperature by analyzing the infrared thermal image. The higher flow rate brings more heat and has the higher cooling rate. Compared with the ice pack that directly contacts with skin, the PEIE-PDMS with the thermal conductivity of 0.134–0.159 W/(mK) can prevent the skin from cold. The temperature can be controlled by the flow rate in real time. To investigate the cooling effect, we attached the microfluidic cooler on the arm of the volunteer (Figure 4D). After 5 min cooling, the average temperature of the cooling area decreased from 35.3°C to 20.3°C when the flow rate of the cold water is 1.75 ml/min. Figure 4E depicts the skin temperature at the various flow rate of cold water. Lower flow rate generates the moderate cooling effect, while the higher flow rate provides the rapid cooling. At the beginning of the pain treatment, higher flow rate may be required. In the middle of the pain relief, flow rate can be reduced to avoid the discomfort caused by the local cooling. In addition to the localized cooling, this soft, wearable microfluidic chip can provide the hot compress by flowing the hot water.

### 2.5 Ultra-stretchable viscoelastic channel

The viscoelastic particle focusing has gained the increasing research interest due to its superior focusing performance and simple channel structure. The viscoelastic focusing has shown its great potentials in the biological field for cell washing (Yuan et al., 2017), yeast separation (Liu et al., 2020), bacterial separation (Liu et al., 2022), microalgal separation (Yuan et al., 2021), plasma extraction (Yuan et al., 2016), and exosome separation (Liu et al., 2017). The particles suspending in the viscoelastic medium experiencing elastic force will be attracted to the minimal shear rate (center of the channel). This is called elasto-inertial focusing. Particle size is the key parameter for viscoelastic focusing. The cut-off size to be focused is determined by the geometry of the viscoelastic channel and the cut-off size cannot be changed once the channel is formed. That is, every viscoelastic microfluidic device has a certain focusing threshold. Since the most viscoelastic microfluidic devices are made of PDMS, the geometries of the devices are difficult to alter due to the lack of elasticity of PDMS (Young’s modulus ∼1 MPa, fracture at break ∼70%). Here, we demonstrate an ultra-stretchable microfluidic channel with the matrix of Ecoflex (Young’s modulus ∼200 kPa, fracture at break ∼900%), which can be stretched to tune the geometry of the viscoelastic channel.

**FIGURE 5:**
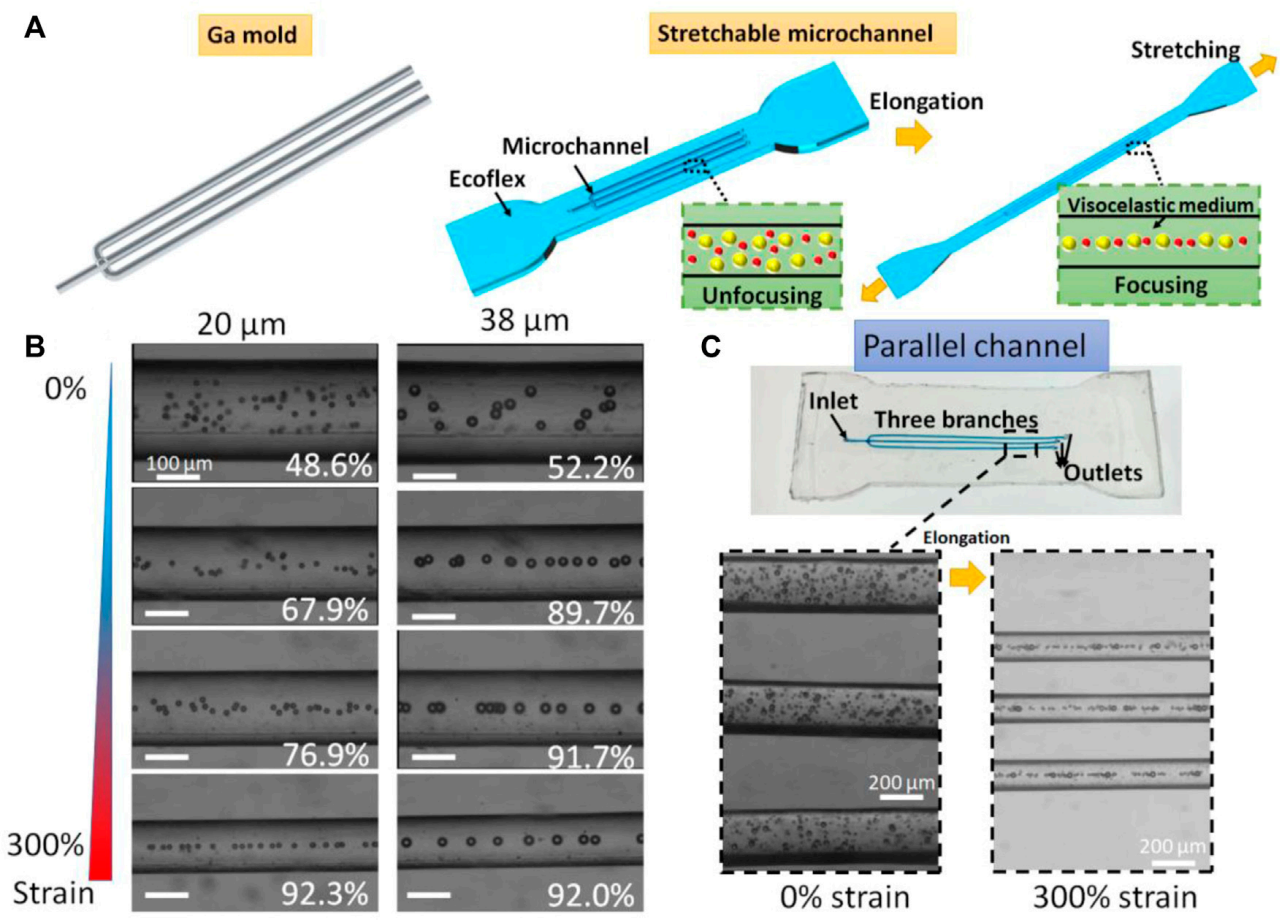
Ultra-stretchable viscoelastic channels. **(A)** Schematic of ultra-stretchable viscoelastic channels that can tune the focusing cut-off size. **(B)** Stacked brightfield images of 20 μm and 38 μm particle migration at different elongations. White numbers represent the focusing efficiencies. **(C)** Parallel channel with three subchannels that can simultaneously manipulate particles for high throughput.

The gallium mold with multiple branches were prepared by the above-mentioned hybrid method. After Ecoflex curing and liquid metal extraction, we obtain a parallel microchannel with one inlet and three outlets. The three branched channels with the same size can simultaneously manipulate particles. The Ecoflex matrix can be highly stretched in which the microchannel size can be modulated simultaneously (Figure 5A). The viscoelastic medium was prepared by adding PEO to the DI water with the final concentration of 1,000 ppm. The 20 μm and 38 μm polystyrene beads were suspended in the PEO solution for the test of the parallel microfluidic channel. The flow rate of the particle sample was set at 60 μL/min. Focusing efficiency is defined as E_n_ = (*w*-*d*/2-*a*)/(*w*-*d*), where *a* represent the width of the focused particle stream, *w* is the channel width, and *d* is the particle diameter. In this work, the particles will be treated as focusing mode when the focusing efficiency is over 80%.

We first studied the viscoelastic particle migration in the single channel (Figure 5B). Focusing efficiencies of 20 μm particles were calculated as 48.6%, 67.9%, 76.9%, and 92.3% for 0%, 100%, 200%, and 300% strain of Ecoflex base, while focusing efficiencies of 38 μm particles were calculated as 52.2%, 89.7%, 91.7%, and 92.0% for the strain ranging from 0 to 300%. Without stretching, both 20 μm and 38 μm polystyrene beads are randomly distributed in the microchannel. By continuously stretching the channel, the 38 μm particles were firstly focused at the strain of 100% and 20 μm particles secondly entered the focusing mode. This enhancement was resulted from the changes in the dimensions including a longer length and a smaller width that lead to a decreased cut-off size of the viscoelastic channel. For three branched channels, the multiple viscoelastic channels could focus particles in a high-throughput manner. The flow rate was set to 180 μL/min. The 20 μm and 38 μm particles were unfocused without stretching. By stretching the Ecoflex matrix, the three channels simultaneously turned longer and narrower (Figure 5C). Both 20 μm and 38 μm particles were in the focusing mode at the stain of 300%. The parallel channels increase the throughput of the viscoelastic channel. More parallel channels can be involved for further improving the throughput of the viscoelastic microchannels. Furthermore, this multiplexing viscoelastic channels can be integrated with imaging flow cytometry (Yan et al., 2020) for high-throughput single-cell analysis.

## 3 Conclusion

In summary, we describe a method to create the 3D microfluidic channel using the LM framework. We develop three different methods to construct the 3D, free-standing LM microstructures by reconfiguring the gallium wires. The solid metal frame is strong enough to stand against gravity. Different encasing materials including PDMS, Ecoflex, PEIE-PDMS was used to cast the LM microstructures. Due to the phase change property of the gallium, the embedded LM microstructures can be melted at low temperature (∼30°C) and extracted from the encasing materials without the damage of the microchannel. Moreover, the encasing materials provide the new functionalities with the 3D microfluidic channels such as ultra-stretchability and stickiness. We also expect that other encasing materials such as ceramics, gels, and castable plastics can be used to extend the functionalities of the microfluidic chip. Two proof-of-concept applications were demonstrated. First, a soft, sticky, on-skin microfluidic cooler is developed based on this process. The microfluidic cooler can have a conformal contact with human skin and provide the focused, minimal invasive cooling power with the temperature control. Second, an ultra-stretchable viscoelastic microchannel with the Ecoflex base is fabricated to continuously tune the viscoelastic particle focusing with a large dynamic range. This work demonstrates a new method for construction of 3D microfluidic channel, suggesting new possibilities for lab-on-a-chip applications and micro total analysis systems.

## 4 Experimental section

Materials: The Ga (99.999% pure), NaOH, PEIE, and PEO (∼2,000,000 Da) were purchased from Shanghai Macklin Biochemical Co., Ltd. Shanghai, China. The PDMS (Sylgard 184, Dow-Corning, United States) and silicone of Ecoflex 00–30 (Smooth-On) were used to prepare the flexible microfluidic devices.

Experiment Setup: Syringe pumps (Legato 100, Kd Scientific, Holliston, MA, United States) were used to inject the cooling water or particle suspension into the channel. The trajectories of particles were recorded by a CCD camera (VEO-E 310L, Phantom, United States). A portable infrared camera FLIR ONE (support for IOS system, MacroPinch Ltd. United States) was used to measure the real-time temperature.

Solution: NaOH solution was prepared by dissolving 100 mg solid NaOH into 25 ml of DI water. For viscoelastic focusing, PEO was dissolved in deionized water (DI water) with a concentration of 1,000 ppm to form the viscoelastic medium.

Softwares: The imaging processing software, ImageJ (open access), was used to stack the brightfield images of particle migration. FLIR Tools (support for IOS system, MacroPinch Ltd. United States) was used for data processing to obtain the average temperature values.

## Data Availability

The original contributions presented in the study are included in the article/Supplementary Material, further inquiries can be directed to the corresponding author.
