# Supplementary material for: A free-standing, phase-change liquid metal mold for 3D flexible microfluidics
Source: Front Bioeng Biotechnol. 2022 Dec 5;10:1094294. doi: 10.3389/fbioe.2022.1094294 (PMC9760860; doi:10.3389/fbioe.2022.1094294)
Supplement: Supplementary file 1 [file Image1.pdf]

Supporting information

for

A free-standing, phase change liquid metal mold for 3D flexible microfluidics

Sheng Yan[1*], Qingwei Yuan[2], Jialin Wu[2], Zixuan Jia[1]
[1]Institute for Advanced Study, Shenzhen University, Shenzhen 518060, China
[2]Nanophotonics Research Center, Institute of Microscale Optoelectronics, Shenzhen University, Shenzhen 518060, China
Corresponding author email: shengyan@szu.edu.cn

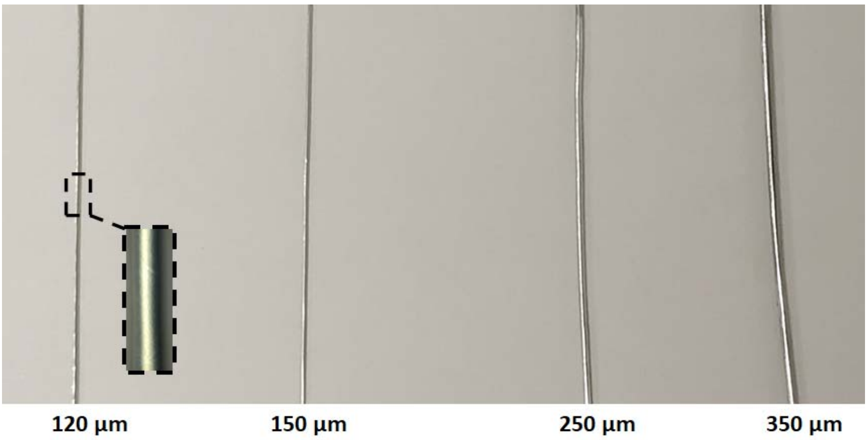

Figure S1 The diameter of Ga wire ranging from 120 μm to 350 μm.

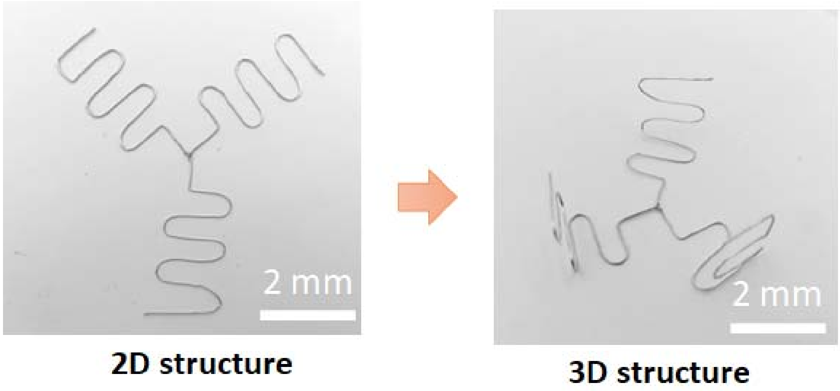

Figure S2 The branched Ga mold can be tuned from 2D to 3D structures.

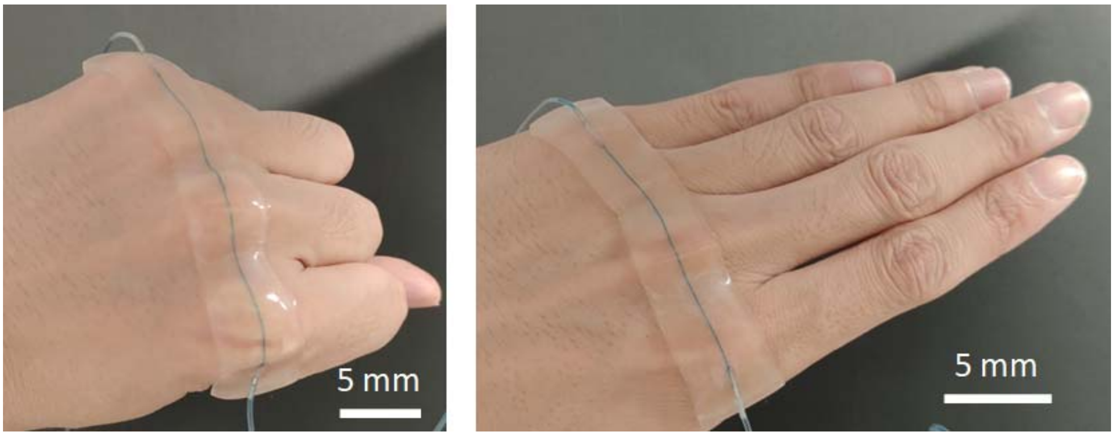

Figure S3 The attachment of the PEIE-PDMS microfluidic chip on the fist. The 1D microchannel turns into 3D microchannel.

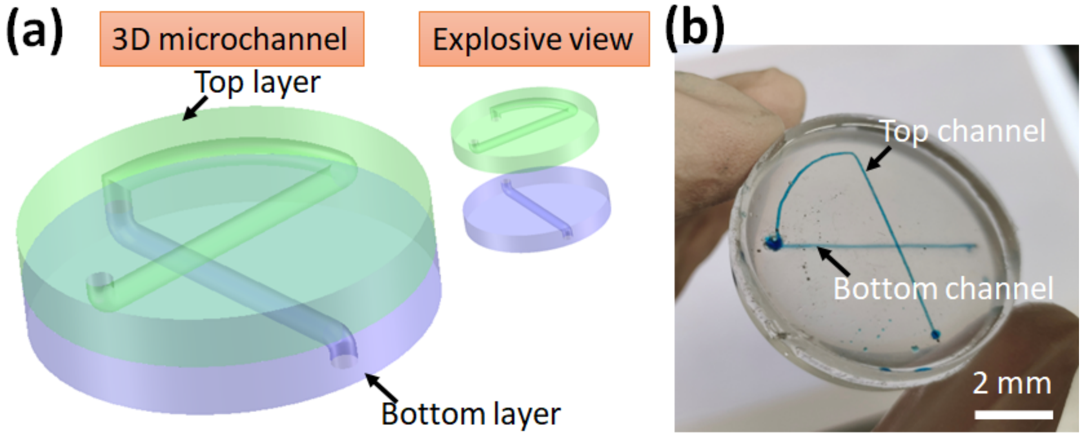

Figure S4 Modular microfluidic device assembled by the two sticky PEIE-PDMS chips. (a) Schematic of assembled 3D microfluidic device. (b) The image of the assembled microfluidic device.
